# Brain activation during processing of angry facial expressions in patients with alcohol dependency

**DOI:** 10.1186/s40101-015-0046-6

**Published:** 2015-03-01

**Authors:** Mi-Sook Park, Sook-Hee Kim, Sunju Sohn, Gap-Jung Kim, Yeon-Kyu Kim, Jin-Hun Sohn

**Affiliations:** Department of Psychology, Chungnam National University, 99 Daehak-ro, Yuseong-gu, Daejeon, 305-764 South Korea; Graduate School of Health and Complementary Medicine, Wonkwang University, 460 Iksandae-ro, Iksan, Jeonbuk 570-749 South Korea; Department of Social Welfare, Cheongju University, 298 Daeseong-ro, Cheongju-si, Chungcheongbuk-do 363-764 South Korea; Department of Psychiatry, Hanmaum Alcohol Treatment Center, 513-1 Jangan-dong, Seo-gu, Daejeon, South Korea; Department of Industrial Design, Graduate school of Integrated Frontier Science, Faculty of Design, Kyushu University, 4-9-1 Shiobaru, Minami-ku, Fukuoka 815-8540 Japan

**Keywords:** Alcoholism, Anger, BOLD, Anterior cingulate cortex, Medial prefrontal cortex

## Abstract

**Background:**

Alcoholism is associated with abnormal anger processing. The purpose of this study was to investigate brain regions involved in the evaluation of angry facial expressions in patients with alcohol dependency.

**Methods:**

Brain blood-oxygenation-level-dependent (BOLD) responses to angry faces were measured and compared between patients with alcohol dependency and controls.

**Results:**

During intensity ratings of angry faces, significant differences in BOLD were observed between patients with alcohol dependency and controls. That is, patients who were alcohol-dependent showed significantly greater activation in several brain regions, including the dorsal anterior cingulate cortex (dACC) and medial prefrontal cortex (MPFC).

**Conclusions:**

Following exposure to angry faces, abnormalities in dACC and MPFC activation in patients with alcohol dependency indicated possible inefficiencies or hypersensitivities in social cognitive processing.

## Background

Alcohol serves many purposes for both social drinkers and individuals with alcohol-abuse disorders. As specified in various alcohol-use scales, these purposes include increasing sociability, overcoming shyness or uneasiness, “joining the group,” forgetting about problems, getting drunk or intoxicated, or simply enjoying the taste. However, for many individuals suffering from alcoholism, the urge to drink can be associated with heightened anger. While some turn to alcohol in an attempt to reduce feelings of anger, others feel justified to drink because someone has made them feel angry. These statements are supported by several studies using valid scales. For example, as measured by several State-Trait Anger Expression Inventory subscales [1,2] and the Profile of Mood States scale [3], individuals who abuse alcohol often present higher levels of anger than “normal” individuals.

Parrott *et al.* [4] found that individuals who drink alcohol and have a tendency toward chronic anger are much more likely to become enraged when drinking alcohol. More recently, Kelly *et al.* [5] followed the progress of more than 1,700 patients with alcohol dependence who were being treated in clinical trials every 3 months for over a year and found that they were more likely to become frustrated and angry compared to individuals without alcohol-abuse disorders. This leaves researchers to question “why” anger plays a critical role in the lives of many individuals with alcoholism and “how” they are different from normal people in processing anger. One explanation is that people with alcoholism may have trouble appropriately expressing their emotion, as hostility or aggression is frequently observed among these individuals [6]. Interestingly, hostile attribution biases are often reflected among individuals with alcohol dependence by their sensitivity to angry faces [7]. Thus, an investigation on how these individuals process angry faces compared to people that do not abuse alcohol may shed light onto important traits of alcohol abusers and may help target problem behaviors associated with a lack of anger management.

To date, we know that various brain regions are related to the perception of anger among healthy people, including the left ventrolateral orbitofrontal, right dorsolateral orbitofrontal, bilateral striate, and bilateral occipitotemporal regions [8]. Moreover, the emotional experience of anger is related to the left anterior insula, an affective division of the anterior cingulate cortex (ACC) [9], while rumination of anger is associated with the medial prefrontal cortex (MPFC) [10]. Salloum and colleagues [7] suggested that abnormalities in brain function, particularly dysfunction of the ACC, among individuals with alcohol dependence in evaluation of angry facial expressions. Salloum *et al.* determined this by presenting faces expressing relatively mild (30%) and average (70%) anger and instructed subjects to choose the intensity of each face. During this task, blood-oxygenation-level-dependent (BOLD) responses were measured, and it was determined that alcohol abusers presented greater activation in the ACC than non-alcohol abusers. However, this is the only study to investigate brain activation in relation to anger-associated stimuli among alcoholics. Moreover, previous studies lacked sufficient evidence to suggest any abnormality in a particular part of the brain with regard to processing anger.

In this study, we focused on the identification of specific brain regions associated with the emotional processing of anger among patients with alcohol dependence. Specifically, the goal was to explain how individuals with alcoholism process angry faces compared to healthy controls by observing BOLD brain responses during evaluation of angry facial expressions.

## Methods

### Participants

Eighteen male patients with alcohol dependency (Mean age: 49.83 years, age range = 39 ~ 60 years) in an in-patient treatment facility voluntarily participated in this study. Specific inclusion criteria were as follows: 1) diagnosed as “Alcohol Dependent” by a psychiatrist based on DSM-IV criteria (American Psychiatric Association, 1994), 2) completion of detoxification, and 3) no history of concurrent psychiatric disorder(s). Although we initially recruited a total of 21 subjects, three individuals showed head motion artifacts in the functional magnetic resonance imaging (fMRI) scan and were hence excluded. With strict monitoring of the in-patient hospital, these individuals continued to stay sober from the time of their entry, with a minimum of 11 days to a maximum of 2,051 days of alcohol abstinence (median = 439.63, SD = 591.95). In consultation with their primary physician, patients on prescribed medication (that is, sleeping pills or anti-craving medication) were required to abstain from such medicines for 14 days prior to scanning.

Sixteen male non-alcoholic volunteers with similar demographics (mean age: 50.06 years, age range = 31 ~ 61 years) were recruited as control subjects from the community via advertisements and flyers. Of the initially 17 recruited controls, one subject showed head motion artifacts during the fMRI and was hence excluded from our analyses. These volunteers did not report any history of impairment in the central nervous system or any psychiatric disease. The control subjects stayed abstinent from alcohol for at least 48 h prior to scanning.

The Korean version of the Alcohol Use Disorders Identification Test (AUDIT-K) [11] was administered to both groups to evaluate alcohol use; individuals that scored 15 points or higher met DSM-IV alcohol-use disorders criteria [12]. The Korean version of the Alcohol Dependence Scale (ADS-K) [13] was also administered to assess the level of alcohol dependency in patients suffering from alcoholism. The time frame of reporting any alcohol-related issues consisted of the 12 months prior to their hospitalization. Demographics and alcohol use of both patient and control groups are shown in Table 1.

**Table 1:** **Demographics and alcohol use of study participants**

**Characteristics**	**Control group (** ***n*** **= 16)**	**Patient group (** ***n*** **= 18)**	***t*** **-value**
	**M(SD)**	**M(SD)**	
Age (years)	50.06 (6.10)	49.83 (6.60)	0.11
Educational level	12.38 (3.57)	10.67 (4.05)	1.30
Family history (%)	0	44.4	3.56**
Number of drinks (day per week)	1.02 (1.55)	4.63 (2.25)	5.37***
Amounts of drinks (drinks per drinking day)	2.86 (2.10)	16.25 (16.08)	3.30**
Maximum number of drinks in a lifetime	8.22 (11.26)	29.77 (24.21)	3.26**
AUDIT-K	6.38 (5.54)	27.89 (9.91)	7.67***
ADS-K	28.05 (5.39)	50.00 (12.85)	6.34***

***P < .001, **P < .01. Note. Means (standard deviations) are represented. One drink = 14 g ethanol. *AUDIT-K* Korean version of the Alcohol Use Disorders Identification Test, *ADS-K* Korean version of the Alcohol Dependence Scale.

### Facial stimuli

We used five pictures of angry facial expressions excerpted from the Japanese Female Facial Expression database (JAFFE) [14]. Lyons *et al*. [14] conducted psychological experiments on 60 Japanese females using JAFFE images where subjects were instructed to use a five-point Likert scale (“1” being “weak” to “5” being “intense”) to rate the intensity of emotions displayed in each image. Pictures of facial expressions with an average rating of 4 points or higher on predominantly angry emotions were used for this study so that the facial expressions were intense enough for subjects to easily recognize anger and to also avoid any instance where subjects might confuse neutral emotions with anger. The person featured in each picture varied across all stimuli so as to parcel out any effect of familiarization. The average intensity of anger expressed in the facial stimuli was 4.51 points (SD 0.23).

### Procedures

Subjects arrived in the laboratory 30 min prior to the experiment, received information about the experimental procedure, and signed the consent form. Afterwards, subjects completed the questionnaires on demographics and alcohol use, followed by a briefing on how to participate in the experiment.

The experimental task consisted of 10 blocks, that is, five fixation blocks and five emotional face blocks (that is, anger, fear, disgust, happiness, and sadness) lasting for 35 s per emotion. The fixation block preceded the emotional face block as a baseline, and a cross-hair (“+”) was presented for the entire duration of the fixation block. During the emotional face block (that is, anger, fear, disgust, happiness, and sadness), five pictures of emotional facial expressions were presented for 7 s each, totaling 35 s per emotion. Subjects were asked to rate the intensity of each facial expression on a five-point Likert scale by pressing corresponding buttons specifically designed for this task. Consequently, the experimental task included five fixation blocks and five emotional face blocks, and lasted 350 s in total. Additionally, we counterbalanced the emotional face blocks to cancel out possible order effects across all participants. The experimental procedure during the entire period was conducted in strict compliance with the University Institutional Review Board.

### Imaging parameters

Imaging was conducted on a 3.0 T whole-body ISOL Technology FORTE scanner (ISOL Technology, Korea) equipped with whole-body gradients and a quadrature head coil. Single-shot echo planar fMRI scans were acquired in 35 continuous slices parallel to the anterior commissure-posterior commissure line. Parameters for the fMRI included the following: repetition time/echo time (TR/TE) were 3,000/30 ms, respectively, flip angle 80, field of view (FOV) 240 mm, matrix size 64 × 64, slice thickness 4 mm, and in-plane resolution 3.75 mm. Three dummy scans from the beginning of the run were excluded to decrease the effect of non-steady-state longitudinal magnetization. T1-weighted anatomical images were obtained with a 3-D FLAIR sequence (TR/TE = 280/14 ms, flip angle = 60, FOV = 240 mm, matrix size = 256 × 256, slice thickness = 4 mm).

### Data analysis

Our main focus was on examining how patients with alcohol dependency responded to angry faces compared to normal controls; therefore, only results for the angry face condition were included in the data analyses. Using SPSS 20.0, an independent *t*-test was performed to compare the perceived level of anger intensity between the two groups. In the fMRI data analysis, brain scans from the fixation condition were compared to those obtained during the presentation of angry faces. The imaging data were analyzed with SPM8 (Wellcome Department of Cognitive Neurology, London, UK). All functional images were realigned with the image taken proximate to the anatomical study by using affine transformation routines built into SPM8. The realigned scans were normalized to SPM8’ s template image that uses the space defined by the Montreal Neurologic Institute, which is very similar to the Talairach and Tournoux stereotaxic atlas [15]. Motion correction was done using sinc interpolation. The functional map was smoothed with a 8-mm isotropic Gaussian kernel prior to statistical analysis. The voxel size was 2 × 2 × 2 mm^3^, resulting from normalization. In order to remove any artifacts resulting from cardio-respiration and other cyclical influences, time series data were filtered with a 240-s high-pass filter.

At the first level, the data were analyzed according to a standard box-car block design, after convolving the BOLD signal with a canonical HRF as modeled in SPM8. The individual first-level analyses of the comparisons of angry faces minus fixations were used for a random effect analysis and mean images for each subject were created. At the second level, mean images were combined in a one-sample *t*-test to assess significant group effects. In agreement with previous studies, we used a threshold of *P* < 0.001 uncorrected, rather than the more rigorous *P* < 0.05 corrected for the entire brain volume [16]. An extended threshold of 20 contiguous voxels was then applied to the activation. In the between-groups analysis, contrast images (angry face condition-fixation condition) were entered into a two-sample *t*-test (32 degrees of freedom). All coordinates derived from the statistical analysis were converted from MNI to the Talairach and Tournoux stereotaxic space [15].

To extract beta values from regions of interest (ROIs) (that is, ACC and MPFC), we selected activated clusters in each ROI using xjView (http://www.alivelearn.net/xjview8/). In SPM, by creating a beta extraction batch file, we loaded the ROI image and assigned directories where beta value files for individual subjects were located. As the beta value could be calculated for each condition, we had beta values for 35 s of the angry face condition and 35 s of the fixation condition in both alcohol and control groups.

## Results

### Behavioral results

The mean (SD) of the intensity scores for control and patient groups were 3.72 (0.53) and 3.34 (0.99), respectively. We found that controls and patients performed similarly in the rating of intensity for the expressions of anger (*t*(32) = 1.37, NS).

### fMRI results

**Figure 1:**
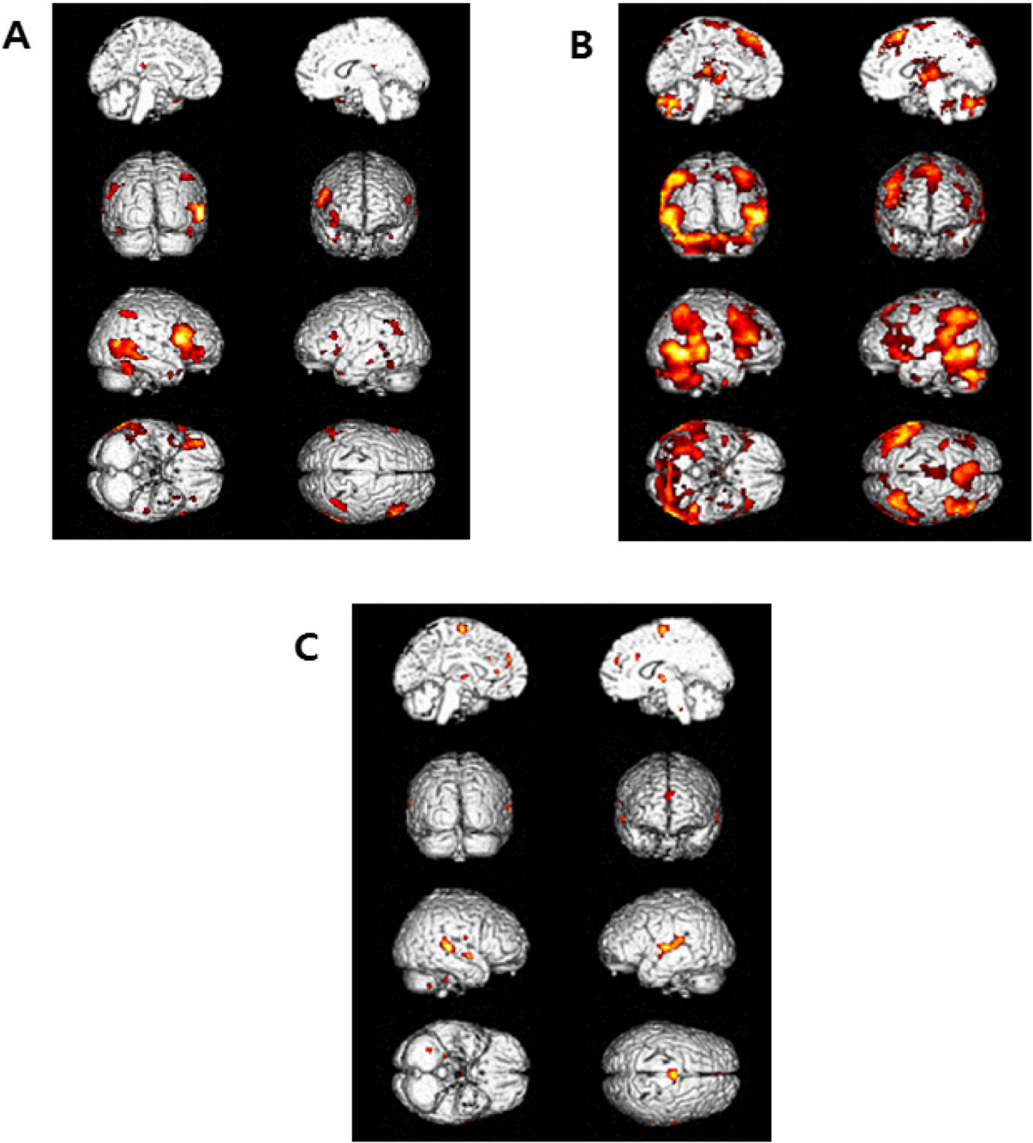
**Brain regions activated during the angry face condition in reference to the fixation condition in each group, and the contrasting effects between the two groups (that is, the patient group versus control group). (A)** the control group (*n* = 16); **(B)** the patient group (*n* = 18); **(C)** contrasting effects of brain activation in the patient group in reference to the control group (uncorrected *P* < 0.001).

Figure 1A presents the brain regions that were activated in the control group during the angry face condition in reference to the fixation condition, while Figure 1B is of the patient group. As for the control group, brain activation was observed in right dorsolateral prefrontal cortex (DLPFC) (Brodmann’s Areas (BA) 46), bilateral culmen, left ventrolateral prefrontal cortex (VLPFC;BA 45)/left lateral orbitalfrontal cortex (LOFC; BA 47), right superior parietal lobule (BA 7), left inferior and middle temporal gyri (BA 20/39), right middle temporal gyrus (BA 21/37), left thalamus, and left superior temporal gyrus (BA 22)/left anterior temporal lobe (ATL; BA 38) (Figure 1A). A stark contrast was observed in the patient group results. That is, activated brain regions were the right superior frontal gyrus (BA 6), left inferior temporal gyrus (BA 20), left middle frontal gyrus (BA 6), left cingulate gyrus (BA 24), right postcentral gyrus (BA 43), right superior and middle temporal gyri (BA 21/38), left uncus/parahippocampal gyrus, and left precuneus (BA 7) (Figure 1B).

Measures of between-group differences provided clarification on the atypical anger-processing pattern seen in patients with alcohol dependency. As shown in Figure 1C, when the brain activation observed between the angry face and fixation conditions of both groups were compared, the patient group exhibited significantly greater activity in the right supplementary motor area (SMA; BA 6), bilateral dorsal anterior cingulate cortex (dACC;BA 32), left medial prefrontal cortex (MPFC; BA 9), right thalamus, and bilateral superior temporal gyri. Talairach coordinates and *t*-scores of each activated area are shown in Table 2.

**Table 2:** **Talairach coordinates and** ***t*** **-scores of activated brain areas**

**Region**	**Side**	**X**	**Y**	**Z**	**Brodmann’s areas (BA)**	***t*** **-value**
Angry face-fixation comparison						
The control group						
Dorsolateral prefrontal cortex	Right	46	16	20	46	5.60
Culmen	Right	46	−46	−28		4.60
	Left	−44	−52	−18		3.40
Ventrolateral prefrontal cortex	Left	−58	22	20	45	4.17
	Left	−44	16	0		3.64
Lateral orbital frontal cortex	Left	−34	34	−2	47	3.72
Superior parietal lobule	Right	40	−56	50	7	4.05
Inferior temporal gyrus	Left	−52	−24	−18	20	3.98
Middle temporal gyrus	Left	−58	−66	24	39	3.66
	Right	46	6	−30	21	3.80
	Right	64	−52	−2	37	5.15
Thalamus	Left	−6	−30	16		3.76
Anterior temporal lobe	Left	−34	10	−32	38	3.64
	Left	−66	−40	6	22	3.34
The patient group						
Superior frontal gyrus	Right	6	18	60	6	6.61
Inferior temporal gyrus	Left	−44	−2	−36	20	4.28
Middle frontal gyrus	Left	−34	0	54	6	4.26
Cingulate gyrus	Left	−20	−12	44	24	4.18
Postcentral gyrus	Right	66	−10	20	43	4.04
Superior temporal gyrus	Right	48	20	−22	38	3.78
Middle temporal gyrus	Right	50	−30	−8	21	8.18
Uncus/parahippocampal gyrus	Left	−24	−2	−26		3.53
Precuneus	Left	−8	−52	62	7	3.08
Contrasted brain activation areas						
The patient group > the control group						
Superior temporal gyrus	Right	62	−32	8	22	4.70
	Right	56	−2	−6	22	3.23
	Right	40	−30	8	41	3.20
Supplementary motor area	Right	4	−10	68	6	3.98
Superior temporal gyrus	Left	−56	−16	6	41	3.68
Medial prefrontal cortex	Left	−2	50	30	9	3.40
Thalamus	Right	6	−10	2		3.30
Postcentral gyrus	Right	64	−8	18	43	3.18
Dorsal anterior cingulate cortex	Left	−10	32	10	32	3.16
Cerebellar tonsil	Right	32	−56	−44		3.12
	Right	24	−32	−38		3.02
Cingulate gyrus	Right	8	22	32	32	2.93

Brain activation comparisons between the angry face and the fixation conditions in each group and contrasting effects between two groups (that is, the patient group versus control group) (uncorrected *P* < 0.001).

**Figure 2:**
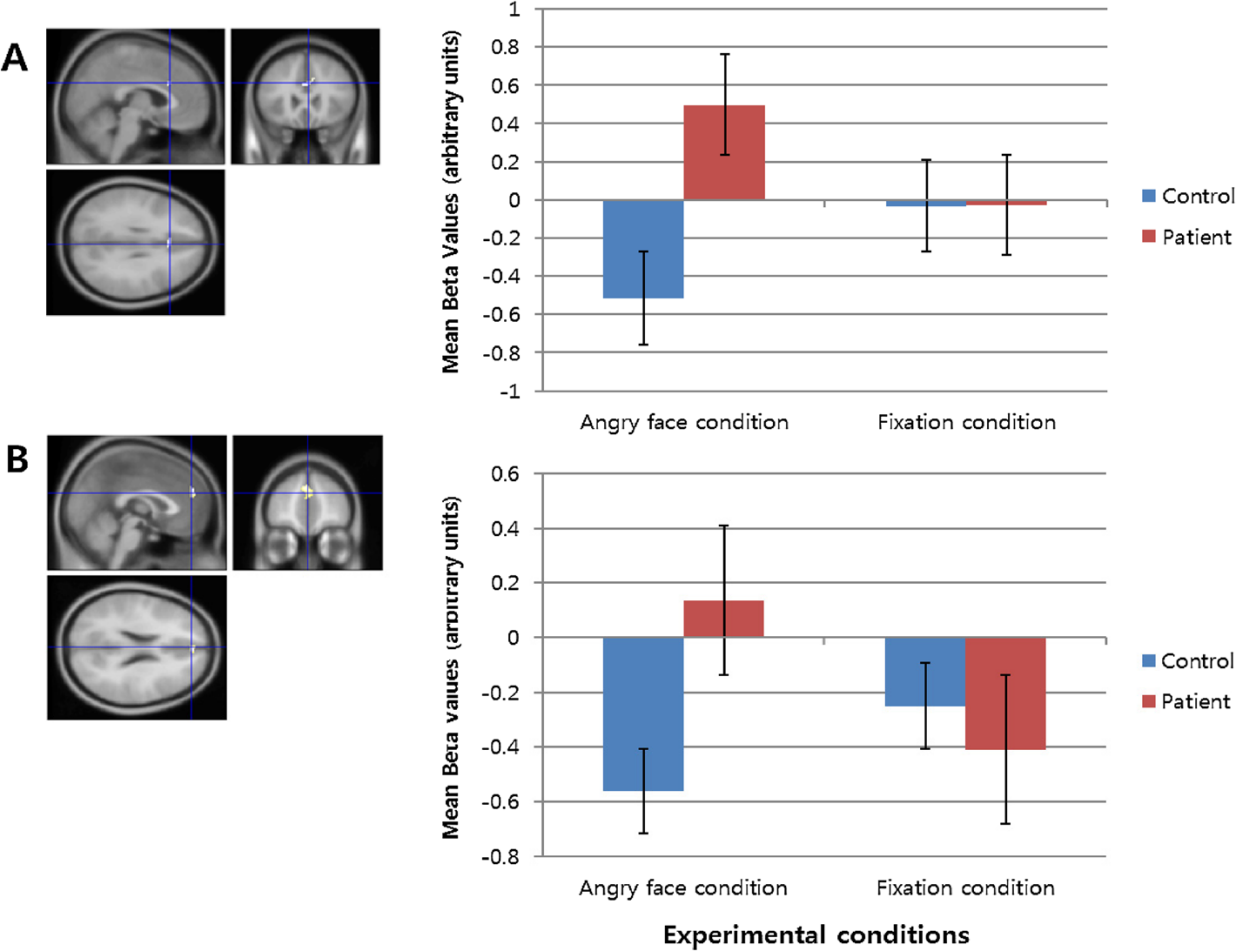
**The extracted beta values for the bilateral dACC (A) and left MPFC (B) (average beta value ± SD) in the control and patient groups for each experimental condition (that is, angry face and fixation conditions).**

Extracted beta values for the dACC and MPFC regions in both groups for each experimental condition (that is, angry face and fixation conditions) are shown in Figure 2A, B. When we conducted small-volume corrections on the ROIs (that is, dACC and MPFC), we were not able to find significant voxels. However, results from an independent *t*-test (SPSS 20.0) with the ROI beta values (the angry face condition - the fixation condition) showed a significant difference between control and alcohol-dependent groups (dACC: *t*(32) = 3.167, *P* < 0.01; MPFC: *t*(32) = 3.316, *P* < 0.01).

## Discussion

The findings of the current study are as follows. In the control group, brain activation was observed during the processing of angry faces in the right DLPFC (BA 46), left VLPFC (BA 45), left LOFC (BA 47), and left ATL (BA 38). This is consistent with what has been observed of the brain regions involved in anger perception or experience [8]. The OFC, in particular, has been shown as a brain area activated when encountered with angry facial expressions among normal people [17], and the results of our control group echo this finding.

Brain activation during anger processing in patients with alcoholism differed from the control group in a number of cortical regions. A finding that draws particular interest is the significantly greater activation that was observed in the bilateral dACC (BA 32) among the patient group during the anger condition. The ACC is involved in attention but is also activated during heightened anger [10]. Interestingly, this region seems to be more involved in anger control than in the actual expression of anger *per se* [18]. For instance, recent reviews of the ACC by Shenhav *et al.* [19] and Gasquoine [20] note that the “ACC contributes to behavior by modifying responses especially in reaction to challenging cognitive and physical states that require additional effortful cognitive control.” This idea is similar to Eisenberger and Lieberman’s notion of the ACC as a “neural alarm system” [21]. Thus, in relation to the current study, ACC activation may be indicative of inefficient functioning and/or hypersensitivity to angry faces in participants that were alcohol-dependent. In other words, participants with alcohol dependency require recruitment of a wider network of regions implicated in cognitive control and self-regulation than healthy controls.

We also found that, during evaluation of angry facial expressions, participants that were alcohol-dependent showed greater activation in the MPFC (BA 9). The greater MPFC activation in patients with alcohol dependency could indicate more rumination [10]. However, because the presentation of angry faces and the subsequent rating of their intensity may not completely elicit rumination, it is plausible to suggest that the MPFC activation may reflect inefficient or hypersensitive social cognitive processing when individuals encounter threatening angry faces. This is because along with its involvement in angry rumination, the MPFC is more broadly associated with social cognition [22].

Despite our findings showing an abnormality in brain functioning when patients with alcoholism are faced with intense anger, they did not differ from the control group in terms of behavioral outcomes on perceived anger intensity. This may be due to the possibility that our stimuli and procedure could not precisely detect the behavioral malfunction of patients with alcohol dependency when processing anger. An alternative explanation could be an issue with the intensity of the stimuli. More specifically, a ceiling effect may have occurred that made it evidently easy for subjects to identify anger, yet not sensitive enough to detect varying degrees of behavioral issues. On the other hand, it is conceivable that individuals with alcohol abuse may have experienced difficulty in self-reporting their perceived level of emotion. In fact, this has been supported by previous studies showing that individuals with alcoholism, despite lower performance scores, report equivalent degrees of difficulty in completing emotional-decoding tasks as did normal controls [23]. Given this issue, the patient group might not have accurately reported their perceived level of anger.

This study has a number of limitations. First, the period of alcohol abstinence varied across study participants (from 11 to 2,051 days), which made it difficult to suggest a relationship between the length of abstinence and their anger perception. Second, our study did not control for possible sleep-deprivation issue among patients that were alcohol-dependent. This may be an arguable issue given that frequent awakening after sleep onset is often seen in patients with alcohol dependency [24]. Moreover, insomnia and/or sleep deprivation have been linked with inaccurate recognition of human emotions, thus, limited ability to control for anger ventilation [25].

Various clinical studies have emphasized hypersensitivity to threats among individuals with alcohol dependence and that these individuals experience intense emotions to negative stimuli. In that sense, despite their limitations, our findings are unique in that greater activation in the dACC and MPFC might suggest inefficient functioning and/or hypersensitivity when patients with alcohol dependency process angry faces.
